# Study protocol for “Moving Bright, Eating Smart”– A phase 2 clinical trial on the acceptability and feasibility of a diet and physical activity intervention to prevent recurrence in colorectal cancer survivors

**DOI:** 10.1186/1471-2458-13-487

**Published:** 2013-05-20

**Authors:** Judy WC Ho, Antoinette M Lee, Duncan J Macfarlane, Daniel YT Fong, Sharron Leung, Ester Cerin, Wynnie YY Chan, Ivy PF Leung, Sharon HS Lam, Aliki J Taylor, Kar-keung Cheng

**Affiliations:** 1 Division of Colorectal Surgery, Department of Surgery, Li Ka Shing Faculty of Medicine, The University of Hong Kong, Pokfulam, Hong Kong; 2 Department of Psychiatry, Li Ka Shing Faculty of Medicine, The University of Hong Kong, Pokfulam, Hong Kong; 3 Institute of Human Performance, The University of Hong Kong, Pokfulam, Hong Kong; 4 School of Nursing, Li Ka Shing Faculty of Medicine, The University of Hong Kong, Pokfulam, Hong Kong; 5 School of Nursing, Hong Kong Baptist Hospital, 6/F, C-Bons International Building 108 Wai Yip Street, Kwun Tong, Kowloon, Hong Kong; 6 School of Exercise and Nutrition Sciences, Faculty of Health, Deakin University, 221 Burwood Highway, Burwood, VIC, Australia; 7 School of Professional and Continuing Education, The University of Hong Kong, Pokfulam, Hong Kong; 8 Department of Dietetics, Queen Elizabeth Hospital, Gascoigne Road, Kowloon, Hong Kong; 9 Department of Public Health, Epidemiology and Biostatistics, University of Birmingham, Public Health Building, Edgbaston, Birmingham B15 2TT, UK

**Keywords:** Colorectal cancer, Cancer survivor, Diet, Meat, Grains, Physical activity, Behavioural intervention, Feasibility, Acceptability, Randomised

## Abstract

**Background:**

Colorectal cancer is the second most common cancer and cancer-killer in Hong Kong with an alarming increasing incidence in recent years. The latest World Cancer Research Fund report concluded that foods low in fibre, and high in red and processed meat cause colorectal cancer whereas physical activity protects against colon cancer. Yet, the influence of these lifestyle factors on cancer outcome is largely unknown even though cancer survivors are eager for lifestyle modifications. Observational studies suggested that low intake of a Western-pattern diet and high physical activity level reduced colorectal cancer mortality. The Theory of Planned Behaviour and the Health Action Process Approach have guided the design of intervention models targeting a wide range of health-related behaviours.

**Methods/design:**

We aim to demonstrate the feasibility of two behavioural interventions intended to improve colorectal cancer outcome and which are designed to increase physical activity level and reduce consumption of a Western-pattern diet. This three year study will be a multicentre, randomised controlled trial in a 2x2 factorial design comparing the “Moving Bright, Eating Smart” (physical activity and diet) programme against usual care. Subjects will be recruited over a 12-month period, undertake intervention for 12 months and followed up for a further 12 months. Baseline, interim and three post-intervention assessments will be conducted.

Two hundred and twenty-two colorectal cancer patients who completed curative treatment without evidence of recurrence will be recruited into the study. Primary outcome measure will be whether physical activity and dietary targets are met at the end of the 12-month intervention. Secondary outcome measures include the magnitude and mechanism of behavioural change, the degree and determinants of compliance, and the additional health benefits and side effects of the intervention.

**Discussion:**

The results of this study will establish the feasibility of targeting the two behaviours (diet and physical activity) and demonstrate the magnitude of behaviour change. The information will facilitate the design of a further larger phase III randomised controlled trial with colorectal cancer outcome as the study endpoint to determine whether this intervention model would reduce colorectal cancer recurrence and mortality.

**Trial registration:**

ClinicalTrials.gov No: NCT01708824

## Background

### Colorectal cancer, treatment outcome and lifestyle factors

According to the Hong Kong Cancer Registry [1], colorectal cancer (CRC) was the second most common cancer and the second highest cancer-killer in Hong Kong with 4,370 new cases and 1,864 deaths in 2010, respectively. Moreover, the crude incidence rate of CRC increased from 48.2 per 100,000 in 2000 to 62.2 per 100,000 in 2010 [1]. CRC may soon become the most common cancer in Hong Kong. This alarming trend is mirrored in many Asian countries [2].

The latest report of the World Cancer Research Fund (WCRF) [3] summarises convincing evidence from published observational studies that physical activity (PA) protects against colon cancer (CC) while foods low in dietary fibre, high in red and processed meat, high levels of alcohol drinking in men, body fatness, abdominal fatness and factors leading to greater adult attained height cause CRC. However, the influence of these lifestyle factors on the outcome of patients with established CRC is largely unknown.

With advances in treatment, CRC survivors are now living longer. Many of them are highly motivated to seek information about lifestyle modifications that improve quality of life and increase their chance of prolonged life and recovery from cancer. Although evidence on certain aspects of PA or diet specifically affecting health outcomes in cancer survivors is emerging, it is not yet sufficiently established to allow firm recommendations to be made [4].

The observational Nurses’ Health Study showed that recreational PA reduced cancer-specific and overall mortality for stage I to III female CRC patients [5], while in another observational study [6], maintaining weekly PA levels over 18 metabolic-equivalent-hours (MET-hours) reduced recurrence and mortality in stage III CC after adjuvant chemotherapy.

A recent observational study compared cancer outcome of stage III CC in two dietary groups [7]. Compared with patients with the lowest mean intake of a Western-pattern diet (i.e., 2.3 servings of red meat weekly, 1.8 servings of processed meat weekly, 2.0 servings of refined grains daily, and less than 1 serving of sugary desserts daily), those with the highest mean intake (i.e., 6 servings of red meat weekly, 5.6 servings of processed meat weekly, 5.8 servings of refined grains daily, and 2.5 servings of sugary desserts daily) had an adjusted hazard ratio for death of 3.25 (95% confidence interval 2.04-5.19). No association was found of a prudent dietary pattern (i.e., high intakes of fruit, vegetable, poultry and fish) with CC mortality and recurrence. Moreover, the two patterns were not inter-correlated (Spearman correlation 0.02).

Our literature review identified a small number of published randomised controlled trials (RCT) or feasibility studies [8-12] on lifestyle intervention of CRC survivors. No CRC intervention has been conducted in an Asian population. Furthermore, to date, there are no published data regarding the effect of PA and/or dietary interventions on CRC outcomes.

### Theories on health behaviour

The Theory of Planned Behaviour (TPB) [13] is one of the most widely tested theories explaining and predicting intentions to perform a wide range of health-related behaviours [14-18]. The TPB has been the framework for the design of intervention models targeted at health behaviour change [19,20] and posits that an individual’s behaviour is predicted by his/her intention to perform the behaviour. This behavioural intention is, in turn, determined by attitudes towards the behaviour, subjective norms, and perceived behavioural control, with each of these being the result of beliefs. The likelihood that an individual’s intention to adopt a health behaviour will therefore be increased if he holds a more favourable attitude towards the behaviour (behavioural beliefs), believes that significant others want him to adopt the behaviour (normative beliefs), and perceives that the behaviour is under his control (control beliefs). The three sets of belief constructs can thus be targeted by interventions for health behaviour change. Useful as the TPB is, it is important to note that intentions to behave do not necessarily lead to actual performance of behaviours. The discrepancy between intended and actual behaviours is termed the intention-behaviour gap [21].

The Health Action Process Approach (HAPA) [22] integrates social-cognitive theory [23], the theory of reasoned action [24] and the volition theories [25,26]. HAPA is particularly useful in guiding the development and design of intervention aimed to enhance health self-regulation [27] that involves motivation, volitional, and actional processes of abandoning health-compromising behaviours and adopting and maintaining health-enhancing behaviours [28]. The model addresses the intention-behaviour gap and defines ways to identify and modify the factors linking intentions with actual behaviour (mediators). The model categorizes health behavioural changes into two stages (processes): the pre-intentional motivation process and the post-intentional volition process, with the first process leading to behavioural intention and the second to actual health behaviours [27]. After a person has an intention to act, he/she needs detailed instructions on how to perform the desired action and the perceived self-efficacy to initiate and to maintain the action. All these require self-regulatory skills and strategies such as action planning [22]. Interventions for changing health behaviours can target attitudes, perceived barriers, personal vulnerability and perceived self-efficacy, however, only stage-matched conditions can bring about benefits on the participants. For example, only intenders and actors will benefit from self-regulatory efforts.

### Study objectives

Based on the TPB and HAPA, this study aims to evaluate the acceptability and feasibility of two behavioural interventions on CRC survivors intended to improve cancer outcome and are designed to increase PA levels and reduce consumption of a Western-pattern diet (i.e., high intake of red and processed meat, dietary fat, refined grains and sugary desserts) [7,29].

## Methods/design

Ethical approval for the study has been received from the Institutional Review Board of the Hong Kong West Cluster, the Hospital Authority in Hong Kong (see later). All participants will receive a written participant information sheet explaining the trial and all will be asked to give written consent prior to participation.

### Study/trial design

This will be a three-year multicentre, randomised controlled trial (RCT) following a 2x2 factorial design comparing PA and dietary interventions with usual care in CRC patients.

A 2x2 factorial design is chosen because it has the advantage of testing both interventions with a smaller sampler size when compared with the alternative of a three parallel group design.

### Treatment period and follow up

Dietary and PA interventions will be delivered over a 12 month period and participants will be followed up for a further 12 months.

Baseline assessment will be conducted at the University of Hong Kong. All baseline measures will be made prior to group allocation. An interim outcome assessment will be performed six months after randomization. Outcome/follow-up assessments will be made at 12, 18 and 24 months after randomization. Staff responsible for outcome assessment will not deliver the intervention. All outcome assessments will be subject to a strict protocol with researchers blinded to group allocation.

All participants (including the usual care group) will be given written, publicly-available general advice that encourages healthy lifestyles.

Participants in the PA intervention group will have one face-to-face intervention contact at the beginning followed by fortnightly telephone contacts for 12 months. They will also receive 12 stage-based information pamphlets and four newsletters by mail and will attend four group meetings during the 12-month intervention period.

Participants in the dietary intervention group will have two face-to-face intervention contacts during the first four months and fortnightly telephone contacts throughout the 12 months. They will also receive 12 stage-based information pamphlets and four newsletters by mail and will attend four group meetings during the 12-month intervention period.

The interventions will be delivered by trained research staff and the research team members.

### Primary outcome

To evaluate the acceptability and feasibility of two behaviour interventions for CRC survivors intended to improve cancer outcome. These interventions are designed to:

1. Increase PA levels to improve general health and cancer outcome;

2. Decrease consumption of a Western-pattern diet.

The PA target for improving general health is 30 minutes of moderate-to-vigorous intensity PA (MVPA) five days a week (equivalent to 10 MET-hours per week). The PA target for improving cancer outcome is 60 minutes of MVPA five days a week (equivalent to 18–20 MET-hours per week).

The dietary targets are to limit weekly red or processed meat intake to <5 servings and to limit daily refined grain intake to two servings.

The primary outcome measure will be whether the PA or dietary targets are met at the end of the 12-month intervention.

### Secondary outcomes

To assess the magnitude of PA and dietary change and estimate the association of such changes with changes in the underlying theoretical constructs (mechanisms of behavioural change).

We will determine:

1. The degree and determinants of compliance to the intervention.

2. The additional health benefits (including body composition, physical fitness, quality of life and mood), and any side effects (including nutritional deficiency and PA-associated injury) of the intervention.

### Measures/assessment instrument

Measures of all outcome points will be completed face-to-face. Details of the outcomes to be collected at different time points and the instruments used are shown in Table 1.

**Table 1:** Outcome measures

	**Measures**	**When**
**Primary outcome**		
PA target – general health	Accelerometer	0 M, 6 M, 12 M, 18 M, 24 M
PA target – cancer outcome	Accelerometer	0 M, 6 M, 12 M, 18 M, 24 M
Dietary target – red/processed meat	FFQ [30]	0 M, 6 M, 12 M, 18 M, 24 M
Dietary target – refined grain	FFQ [30]	0 M, 6 M, 12 M, 18 M, 24 M
**Secondary outcome**		
Magnitude of PA change	Accelerometer	0 M, 6 M, 12 M, 18 M, 24 M
	GPAQ [31]	
Magnitude of dietary change	FFQ [30]	0 M, 6 M, 12 M, 18 M, 24 M
Compliance	Intervention record; pedometer, food diary	6 M, 12 M
Measurement of theoretical constructs	Questionnaire	0 M, 6 M, 12 M, 18 M, 24 M
Facilitators and barriers of intervention	Questionnaire	6 M, 12 M
	Focus-group discussion	Towards end of intervention (last group meeting)
BMI, WHR	Calibrated scales, stadiometer; tape measure	0 M, 6 M, 12 M, 18 M, 24 M
Body and visceral fat	Bioelectrical impedance	0 M, 12 M, 24 M
Physical fitness	Six-minute ergometry	0 M, 12 M, 24 M
Quality of life	SF12 [32,33], FACT [34]	0 M, 6 M, 12 M, 18 M, 24 M
Mood	HADS [35,36], PSS [37]	0 M, 6 M, 12 M, 18 M, 24 M
Dietary deficiency – caloric and protein intake	FFQ [30]	0 M, 6 M, 12 M, 18 M, 24 M
Dietary associated anaemia	CBC by blood test	0 M, 6 M, 12 M, 18 M, 24 M
PA- associated injury	Direct questioning during phone call	Fortnightly during intervention, 6 M, 12 M

Abbreviations: M-months post-randomization, PA-physical activity, BMI-body mass index, WHR-waist-hip ratio, FFQ-food frequency questionnaire, GPAQ-global physical activity questionnaire, SF12-Short Form 12 item, FACT-Functional Assessment of Cancer Therapy, HADS-Hospital Anxiety and Depression Scale, PSS-Perceived Stress Scale, CBC-complete blood count.

### Centre and participant selection

Participants will be recruited from the surgical and clinical oncology departments of four public hospitals (Queen Mary Hospital, Pamela Youde Nethersole Eastern Hospital, Princess Margaret Hospital and Yan Chai Hospital) in three regions of Hong Kong. The intervention will be delivered in the three regional centres (Queen Mary Hospital on Island West, Pamela Youde Nethersole Eastern Hospital on Island East and Princess Margaret Hospital at Kowloon West). The clinical collaborators of each site have established that the colorectal and oncology teams are willing to participate in this trial.

### Inclusion criteria

Histologically proven colorectal adenocarcinoma patients above the age of 18 years within one year of completion of main cancer treatment who are able to provide informed consent.

### Exclusion criteria

CRC patients who have persistent or recurrent disease at the time of the recruitment, are receiving cancer treatment, suffer from hereditary CRC syndrome(s), have known contraindication to PA, are unable to read Chinese, have intakes of red/proceed meat less than five servings per week and refined grains less than two servings daily, or accumulate more than 300 minutes per week of MVPA.

### Participant recruitment

Patients will be identified from the CRC Case Management Programme conducted in each participating hospital. Cancer diagnosis and treatment status will be confirmed by medical record review.

Potentially eligible participants will be mailed an introductory letter containing information about the study, followed by an initial contact (phone or in person at the clinic) by the Cancer Case Manager of each participating hospital. Patients who express their interest will then be approached by the research staff for further screening of eligibility and informed consent if deemed eligible.

### Informed consent

Those who state their interest in taking part will be given any further information they require and, if eligible, invited to the participating hospital to provide informed consent.

### Registration

Data on individuals invited to participate in the trial, whether they consent to be contacted by the research staff, and their eligibility will be kept by the Cancer Case Managers who initially contacted the potential participants. The research staff will also keep a log of any individuals who decline at the trial consent meeting. A computerized case report form will be completed for all consenting individuals. Details of a primary caregiver (if applicable) will also be collected to facilitate participant follow-up. The research staff will use the trial database to enter and store data on all eligible individuals. Recruitment information will also be monitored at regular intervals by comparing this to the numbers being approached and the numbers declining.

### Withdrawal and loss to follow-up

Individuals have the right to withdraw consent for participation in any aspect of this trial at any time. Their medical care will not be affected at any time by declining to participate in or withdrawing from the trial.

The research team will make every effort to minimize the loss to follow-up. If a participant misses one follow-up, we will try to re-arrange with them a session on at least two further occasions. Participants will receive a travel allowance for attending baseline and outcome assessment at the study centre (the University of Hong Kong).

### Trial intervention

#### The intervention groups

“Moving Bright, Eating Smart” is a personalised, multiple-contact intervention programme based on the TPB [13] and the HAPA [22] which guide interventions on health self-regulation that involve motivation, volitional and actional processes for adopting and maintaining health-enhancing behaviours [22,27]. The HAPA addresses the intention-behaviour gap and aims to identify and modify the mechanism of behaviour change (mediators). The objectives/milestones for each stage of change are shown in Table 2.

**Table 2:** Milestones for each HAPA stage of change

**HAPA stage**	**Milestones**
Pre-intentional stage	1. Change in attitude regarding behaviour change
	2. Perceive behavioural health-link
	3. Perceive social pressure for behavioural change from significant others
Intentional stage	1. Intention to modify behaviour
	2. Perceive behavioural control
	3. Develop optimistic belief about ability to deal with barriers
Actional stage	1. Goal setting and review of behavioural goals
	2. Action planning
	3. Self-monitoring of performance
	4. Feedback
	5. Develop coping strategies to deal with barriers
	6. Behaviour maintenance (relapse prevention and recovery)

Abbreviations: HAPA-Health Action Process Approach.

### Physical activity intervention

The PA targets are:

a. General health target of 30 minutes of MVPA 5 days a week (10 MET-hours per week)

b. Cancer outcome target of 60 minutes of MVPA 5 days a week (18–20 MET-hours per week).

During the first six months, participants will be asked to increase PA progressively to achieve the general health target with the aim of progressing towards the cancer outcome target in the next six months. Our previous unpublished research (Phase 0) indicated that the two most preferred modes of PA intervention would be home-based exercise and an incidental active lifestyle.

The PA intervention will consist of:

(1) One face-to-face motivational interview with emphasis on instilling the belief in health and cancer outcome improvement by increasing PA level, goal setting, exploring PA options, exploring perceived facilitators and barriers to PA change and demonstrating the use of a pedometer for progress monitoring;

(2) Fortnightly motivational phone calls for progress monitoring, providing encouragement and reinforcement as well as problem solving;

(3) Monthly HAPA-stage-of-change matched pamphlets by mail providing information, practical tips and suggested task to consolidate PA change;

(4) Quarterly newsletters by mail for experience sharing among participants;

(5) Quarterly group meetings for promoting social support, demonstrating various PA options and facilitating discussion on barriers to PA change.

All PA participants will be given a pedometer and a monthly PA log as means of monitoring changes in PA level.

### Dietary intervention

The dietary targets are:

(a) Less than five servings of red/processed meat weekly; less than two servings of which will be processed meat;

(b) Two servings of refined grains daily.

During the first six months, participants will be asked to gradually reduce red/processed meat first, followed by refined grains. They will be encouraged to replace red/processed meat with other protein sources and refined grains with wholegrain. In order to establish and enhance perceived self-efficacy to maximize the probability of desired behavioural change, a staggered approach will be adopted, focusing on reduction of red/processed meat first. Strategies to reduce refined grain intake will be initiated within one month after a participant becomes an “actor” of reduced red/processed meat. During the next six months, participants will be expected to progress towards the dietary targets as stated above.

Similar to the PA intervention, the dietary intervention will consist of:

(1) Two face-to-face motivational interviews, one for red/processed meat and another for refined grains;

(2) Fortnightly motivational phone calls;

(3) Monthly HAPA-stage-of-change matched information pamphlets by mail;

(4) Quarterly newsletters by mail;

(5) Quarterly group meeting.

All participants in the dietary group will be given a food diary with monthly dietary logs for monitoring changes in the intakes of red/processed meat and refined grains. A set of eating utensils will also be given to facilitate portion size estimation.

### PA and dietary intervention

This group will receive, where possible, an integrated version of both intervention components. This integrated approach is necessary to avoid intervention overload (participant fatigue) in this particular group.

For all subjects in the intervention groups, the exact content and the pace of the intervention delivered will depend on the participants’ extant PA and dietary pattern, medical co-morbidities, HAPA stage and individual preferences.

### The usual care (control) group

Similar to those in the intervention groups, the usual care participants will be mailed, at regular intervals, five pamphlets containing general advice encouraging a healthy lifestyle, including eating a wide variety of food, eating more fruit and vegetable, increasing physical activity level, maintaining a normal body weight, quitting smoking and avoiding alcohol abuse. The above mentioned information is widely available in the public domains (websites of the World Health Organization and the Department of Health in Hong Kong).

Maintaining contact with these participants from the baseline assessment to the various outcome assessments is done to minimize loss to follow-up. Furthermore, this practice would ensure that all participants receive some lifestyle advice that at the moment is given on an ad hoc basis. We anticipate that the effect of these pamphlets alone would be small.

**Figure 1:**
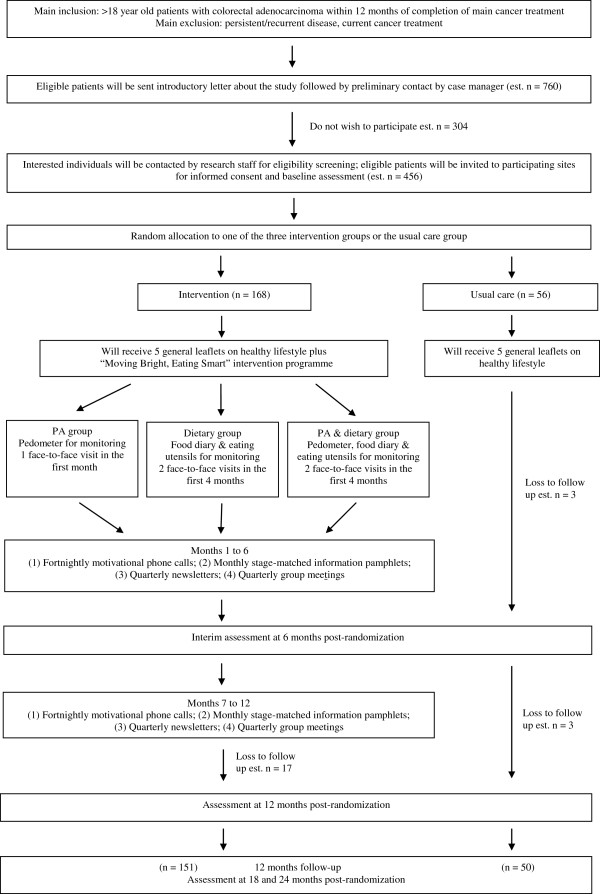
Participant flow chart.

Figure 1 illustrates the participant pathway throughout the trial.

### Serious adverse events and adverse events

No serious adverse events (SAE) are anticipated. However, if any SAE occurs, this will be reported to the Institutional Review Boards and will also be drawn to the immediate attention of the clinical collaborators of the participating departments.

Participants receiving the dietary intervention will be monitored to prevent specific nutrient deficiency including inadequate caloric and protein intakes and iron-deficiency anaemia due to inadequate red meat intake. Participants receiving the PA intervention will be monitored for PA-associated injury.

### Statistical considerations

#### Randomisation

A randomisation schedule will be generated by block randomisation with stratification by study centre and stoma status. The block size will be decided and securely kept by a randomiser who will not be involved in subject recruitment. The randomisation will be managed centrally by the randomiser. When an eligible patient consents to participate in the study, the recruiter will obtain the treatment code from a centralised access restricted randomisation system.

### Sample size

In the control arm, we do not expect that a sizable percentage of participants will achieve the behavioural targets. Nevertheless, we conservatively assume that 10% of them will reach the targeted levels at one year, which results in a larger required sample size to detect the intervention effects. Although we are optimistic about the intervention effect, based on our clinical experience, we consider that a 10-20% improvement in the intervention arm over the usual care would be regarded as minimally significant for promoting the use of the intervention. Hence, we assume an effect size of 15%. To achieve 80% power with a 5% false positive error rate to detect this effect size by a chi-square test, we need 50 subjects per cell and thus 200 patients in total. Expecting a 10% dropout rate, we will need to recruit 224 patients for the study. The sample size should be sufficient when the improvement is larger than expected.

### Analysis

#### Main analysis

Efficacy of the dietary and PA interventions in helping patients to reach the corresponding behavioural target will be assessed by logistic regression analysis. Model adequacy will be examined by the Hosmer-Lemeshow goodness of fit test. The analysis will be performed on two analysis sets, full and per-protocol, to examine the sensitivity of results due to non-compliance. The full analysis set includes all patients as randomised. Patients with missing values will be taken as if they did not reach the targeted level. The per-protocol set includes all patients who complied with the intervention and have no missing values. Conclusions will be made from the results derived from the full analysis set, which is consistent with the intention-to-treat principle. All analysis will be adjusted for recruitment sites and stoma status.

Analysis of secondary outcomes will be performed by mixed-effects analysis to account for extra covariance from repeated measurements taken from a participant.

A 95% confidence interval will accompany all effect estimates and a 5% level of significance will be used in all significance tests.

### Qualitative analysis

Focus-group discussions will be conducted in the last quarterly group meeting investigating the facilitators and barriers to both interventions. The discussion will be audio-taped and contents will be transcribed. Data will be systematically coded using content analysis and analysed by comparing discussion of similar opinions to form themes and at the same time note the deviant opinions from the themes.

### Data storage and retention

Data management will be handled by the Department of Surgery of the University of Hong Kong with data being held according to the International Conference on Harmonisation (ICH) Good Clinical Practice (GCP) E6 Guideline and the Declaration of Helsinki. Data will be held for a minimum of ten years from the completion of the project.

### Ethical approval, research governance and data access

Ethical approval was obtained from the Institutional Review Board of the Hong Kong West Cluster, the Hospital Authority in Hong Kong (Reference number: UW 12–478) with other participating centres providing site-specific approval (Island East reference number: HKEC-2012-068; and Kowloon West reference number: KW/EX-13-002(59–02)). The trial has been registered at the ClinicalTrials.gov with the trial number of NCT01708824.

### Study/trial sponsorship

The University of Hong Kong is the sponsor of this trial.

## Discussion/rationale for the current study

This is the first PA and dietary intervention on CRC survivors in an Asian population.

To date, there is insufficient information available from the published literature as to the most effective way in promoting lifestyle changes in CRC survivors. Moreover, none of these interventions have been conducted in an Asian population where the culture, eating habit, built environment and climate is typically different to most Western populations. This knowledge gap shows the importance of our study.

The latest WCRF recommendation states that cancer survivors should follow the same lifestyle recommendations for cancer prevention. Our previous survey of 150 CRC survivors (unpublished data) showed that <4% of our patients were current drinkers and their mean body mass index and waist-hip ratio were 23.8±3.5 kg/m^2^ and 82.3±10.2 cm, respectively. These findings accentuate the importance of testing the effect of changing PA and diet as they are likely to be the only two modifiable factors worthy of consideration for intervention in our patient population.

In our study, we have chosen two PA targets: the general health target which corresponds to the general public health PA guideline of 10 MET-hours per week; and the cancer outcome target based on the study by Meyerhardt et al. [6] which suggested that a PA level of more than 18 MET-hours per week was required to significantly reduce cancer mortality. The on-going CHALLENGE trial [38] on CRC survivors also uses 20 MET-hours per week as the PA target at 6 to 12 months of intervention. Having two PA targets varying in difficulty and with corresponding health benefits may provide encouragement to those participants lacking self-efficacy for, or ability to, change. Besides, a more recent study has noted measurable cancer risk reductions associated with 6 to 12 MET-hours per week of PA [39]. Therefore, given that the evidence is rather inconclusive and the possibility of lower levels of PA being sufficient to significantly improve post-diagnosis cancer outcomes, it is important to evaluate the feasibility of meeting this more moderate behavioural target. Furthermore, engagement in 10 MET-hours per week has been associated with significant reduction in overall mortality risk [40], risk of a range of non-communicable diseases [41,42] and better quality of life in cancer survivors [43]. These are important non-trivial health outcomes for CRC survivors as much as they are for the general population.

The study by Meyerhardt et al. [7] showed that the lowest intake of a Western-pattern diet significantly reduced cancer mortality when compared to the highest intake. Our previous survey (unpublished data) concluded that <1% of the 150 CRC survivors studied consumed more than one serving of sugary desserts daily. Therefore, the rounded-up lowest intake levels of red/processed meat (5 servings per week) and refined grains (2 servings daily) were adopted as our dietary target.

The main proposed mechanism of PA and diet influencing CRC outcome relates to energy balance [29]. Physical inactivity and a Western-pattern diet shift energy balance leading to hyperinsulinaemia, high insulin-like growth factor 1 (IGF-1) levels and insulin resistance [44-47] which stimulate growth and inhibit apoptosis of micro-metastases [47-49] leading to cancer recurrence and mortality [29]. The luminal effect of carcinogens derived from red and processed meat is another possible mechanism associated with colorectal carcinogenesis. With reduced intake of red/processed meat, the reduced luminal effect of carcinogen may result in the reduction of local recurrence, especially for rectal cancer. Other potential mechanisms relating to PA and diet are alteration in vitamin D, hormonal changes, inflammation and immune modulation [29].

Our planned study follows the Medical Research Council (MRC) framework for the design and evaluation of complex interventions [50]. The design of this Phase 2 trial is based on the TPB and HAPA as well as the findings of our previous work including those from a literature review [8], qualitative interviews and survey. The exact strategies to be adopted and the pace of the intervention to be delivered will depend on the HAPA stage of an individual subject, which will be determined monthly throughout the 12-month intervention.

The results of this Phase 2 trial will be key in establishing the feasibility of targeting the two behaviours (PA and diet) and demonstrating the magnitude of behaviour change. Such information will be essential in the design of a subsequent larger and definitive Phase 3 RCT with CRC outcome as a primary endpoint. By following the MRC framework in designing and evaluating our intervention systematically, we are in the best position to determine its effectiveness in promoting lifestyle changes in CRC survivors, and to determine whether this intervention would be effective in improving CRC outcome.

## Abbreviations

CC: Colon cancer; CRC: Colorectal cancer; GCP: Good clinical practice; HAPA: Health Action Process Approach; ICH: International Conference on Harmonisation; MET: Metabolic equivalent; MRC: Medical Research Council; MVPA: Moderate-to-vigorous intensity physical activity; PA: Physical activity; RCT: Randomised controlled trial; SAE: Serious adverse event; TPB: Theory of Planned Behaviour; WCRF: World Cancer Research Fund.

## Competing interests

The authors declare that they have no competing interests.

## Authors’ contributions

JH conceived of the study, participated in its overall design, obtained funding, coordinated the research team and drafted the manuscript. AL participated in the study design and determined the behavioural theories and strategies to be adopted. DM participated in the study design and determined the content of physical activity intervention and its assessment. DF participated in the study design, determined the study sample size and method of statistical analysis, and supervised the randomization process. SL participated in the study design and determined the behavioural theories and strategies to be adopted. EC participated in the study design, advised on behavioural strategies to be adopted, and determined the content of physical activity intervention and its assessment. WC participated in the design of dietary intervention and assessment. IL participated in the design of dietary intervention and assessment. SHL participated in the design of dietary intervention and assessment. AT conceived of the study and participated in the overall study design. KC conceived of the study, obtained funding and supervised the overall study design. AL, DM, DF, SL, EC, WC, IL, SHL, AT and KC revised the manuscript critically for important intellectual content. All authors read and approved the final manuscript.

## Pre-publication history

The pre-publication history for this paper can be accessed here:

http://www.biomedcentral.com/1471-2458/13/487/prepub
